# COVID-19 vaccine hesitancy in Spain and associated factors

**DOI:** 10.3389/fpubh.2023.1129079

**Published:** 2023-03-16

**Authors:** Maria Falcon, Carmen Rodríguez-Blázquez, María Romay-Barja, Alba Ayala, Alfredo Burgos, María José De Tena-Dávila, Maria João Forjaz

**Affiliations:** ^1^Legal Medicine Department, Biomedical Research Institute (IMIB), University of Murcia, Murcia, Spain; ^2^National Epidemiology Center, Instituto de Salud Carlos III, Madrid, Spain; ^3^National Center of Tropical Diseases, Instituto de Salud Carlos III, Madrid, Spain; ^4^Centro de Investigación Biomédica en Red de Enfermedades Infecciosas (CIBERINFEC), Madrid, Spain; ^5^University Institute on Gender Studies, University Carlos III, Getafe, Spain; ^6^Research Network on Chronic Diseases, Primary Care, and Health Promotion (RICAPPS), Madrid, Spain; ^7^Digital Health Research Unit, Instituto de Salud Carlos III, Madrid, Spain

**Keywords:** COVID-19, vaccine, hesitancy, public health, behavioral insights

## Abstract

**Introduction:**

The present study explores the reasons of those who have not been vaccinated in the later stage of the vaccine rollout in Spain and its associated determinants.

**Methods:**

Cluster and logistic regression analyses were used to assess differences in claimed reasons for vaccine hesitancy in Spain using two samples of unvaccinated people (18–40 years old) gathered by an online cross-sectional survey from social networks (*n* = 910) and from a representative panel (*n* = 963) in October-November 2021.

**Results:**

The main reasons for not being vaccinated were believing that the COVID-19 vaccines had been developed too fast, they were experimental, and they were not safe, endorsed by 68.7% participants in the social network sample and 55.4% in the panel sample. The cluster analysis classified the participants into two groups. Logistic regression showed that Cluster 2 (individuals who reported structural constraints and health-related reasons such as pregnancy or medical recommendation) presented a lower trust in information from health professionals, had a lower willingness to get vaccinated in the future, and avoided less social/family events than those in Cluster 1 (reasons centered in distrust on COVID-19 vaccines, conspiracy thoughts and complacency).

**Conclusions:**

It is important to promote information campaigns that provide reliable information and fight fake news and myths. Future vaccination intention differs in both clusters, so these results are important for developing strategies target to increase vaccination uptake for those who do not reject the COVID-19 vaccine completely.

## 1. Introduction

Spain is one of the leading countries in COVID-19 vaccination adherence. Starting in late December 2020, the country accelerated its vaccination in early 2021, surpassing countries that had made better progress earlier on, such as the USA and the UK (1).

Spain meets the EU objectives in its vaccination strategy (2) with the goal of reducing morbidity and mortality, prioritizing vaccination of the most vulnerable groups and guaranteeing vaccine access and safety. The COVID-19 vaccination campaign in Spain has been considered a success (3), with a much lower percentage of people declining to be vaccinated than other occidental countries.

COVID-19 vaccine acceptance has been monitored from the early stages of the pandemic with the COSMO-Spain study (4) whose results showed an increase of willingness to be vaccinated once the vaccination campaign began, reaching a 94% of vaccine acceptance in October 2021 (https://portalcne.isciii.es/cosmo-spain). This success is multifactorial and could be related to the population's trust in the Spanish national health system, which provides universal healthcare, a long tradition of vaccine compliance and that anti-vaccine advocacy groups have not been as relevant as in other countries (5).

At the time of this study (November 2021), more than 75 million doses of COVID-19 vaccines had been administered in Spain. About 38.2 million people, 80.69% of the Spanish population, had received at least one dose, and more than 37.5 million, 79.1% of the population, had already received the full schedule. In addition, 3.8 million people had received one additional booster dose (6). Nonetheless, according to official statistics almost 7 million people were not vaccinated despite vaccination was available and recommended for them (6). The percentage of the unvaccinated population varies according to the age ranges, with people aged between 18 and 40 years having a lower vaccination rate (6).

Addressing factors influencing COVID-19 vaccine acceptance in population subgroups with low vaccine uptake is of paramount importance. It is necessary to know the viewpoints of people who are hesitant to COVID-19 vaccination, so that interventions to increase vaccination rate can be tailored to the characteristics and reasons of this population.

Vaccination acceptance is a behavior resulting from a complex decision-making process influenced by a wide range of factors (7). The hesitancy of people to be vaccinated is not new and was present before the COVID-19 pandemic. The SAGE Working Group on Vaccine Hesitancy defined it as a “delay in acceptance or refusal of vaccination despite availability of vaccination services” (7). Main determinants of vaccine hesitancy were grouped in the “3Cs” model as Complacency, Convenience and Confidence (7). Complacency entails low risk perception of the disease (7); Convenience or constraints include the physical and psychological barriers to vaccination (8) and Confidence comprises the perception of safety and efficacy of vaccines and the trust in the system in charge of the delivery.

Other factors associated with COVID-19 vaccine hesitancy have been identified, such as health literacy and sociodemographic factors including in addition to age, gender or education level (9–12). There is also an heterogeneous group of reasons for low uptake of COVID-19 vaccines, related to belief in conspiracy theories such as COVID-19 vaccines modify DNA and the speed of development of COVID-19 vaccines; concerns about long term effects, side effects, and unknown future effects on health; or worries related to fertility, pregnancy, and breastfeeding (9, 13, 14).

The present study sought to explore the reasons of those who have not been vaccinated in the later stage of the vaccine rollout in Spain, when COVID-19 vaccines were available for the full adult population, as well as to describe the profile and characteristics of non-vaccinated people and its associated determinants.

## 2. Material and methods

### 2.1. Study area and population

This cross-sectional study was carried out in October-November 2021. The survey aimed to assess the reasons for not being vaccinated of COVID-19 in Spain, together with the risk perception, preventive practices, trust on different information sources and health literacy of the unvaccinated population.

### 2.2. Sampling and data collection

Two different sampling methods were implemented to ensure access to the target population. First, the Spanish population older than 16 years was invited to participate in an online survey disseminated through social networks (WhatsApp, Twitter, LinkedIn, and Facebook) from October 1 to 19th 2021. A non- probabilistic method using a snowball sampling technique was used to reach the participants. The survey was posted on the researchers' social media profiles and sent by WhatsApp with a standard message (“You haven't been vaccinated? We want to hear from you!” https://encuestas.isciii.es/index.php/686837) inviting the population to participate and encouraging them to share the survey link with their contacts. Several national free newspapers echoed the initiative and published the news, including the link to the survey. The invitation link received 5.902 hits, but 4.178 people did not complete the survey and 372 questionnaires presented errors or inconsistencies. Out of the 2.312 fulfilled questionnaires, 1,998 participants were unvaccinated. Only respondents between 18 and 40 years old (*N* = 910) were included in this analysis (Social networks sample).

At the time of this survey, the number of COVID-19 cases in Spain was 19.884, with a cumulative incidence of 41.90 in the last 14 days (15). The percentage of the population older than 12 years with at least one dose of the vaccines was 90%. The percentage of vaccinated people in the age range between 18 and 40 years old was lower, around 80% (16).

In November 2021, we launched a panel survey with the same questionnaire through a consumer research company matching the Spanish general population in terms of education, gender and area of residence. Participants' age was restricted to the group of 18 to 40 years old. This sample was weighted, with an efficiency of 76.79% and a sampling error of 3.02%. The invitation to complete the panel survey was sent to 19.424 people aged 18–40 years, and 1.775 people who had not been fully vaccinated accepted. Of these, 1.051 participants completed the survey in a valid way, and 963 had not received any vaccination dose (Panel sample).

By the third week of November 2021, the number of COVID-19 cases had increased to 66.004 (17), and more than 75 million doses of COVID-19 vaccines had been administered in Spain. 90.8% of the population older than 12 years had received at least one dose of the vaccines, but people between the ages of 18 and 40 continued to have lower vaccination rates (80%) (6).

The Ethics Committee of the Institute of Health Carlos III approved both studies (CEI PI 61_2021-v2 and CEI PI 59_2020-v2_Ampliación 2021-v2) and participants signed the informed consent.

### 2.3. Variables

This study is part of a larger project, the COSMO-SPAIN Project (https://portalcne.isciii.es/cosmo-spain) (4), based in the COVID-19 Snapshot Monitoring WHO initiative to conduct behavioral insights studies related to COVID-19 (18). The survey items included in the COSMO-WHO survey tool, originally in English, were translated by professional translators and adapted by the COSMO-SPAIN team.

The questionnaire gathered information about sex (male, female), age, education, living with older people (yes, no) and employment situation.

To explore participants' motives, we used previously stated reasons for hesitation about COVID-19 vaccines (9, 13). Respondents could choose multiple answers from a list of 18 potential reasons and an open option.

Future intention to be vaccinate against COVID-19 was asked “Do you think you will get vaccinated in the future?” (Yes, No, I don't know). Risk perception was measured with the question “How severe would contracting the coronavirus/COVID-19 be for you?” answered in a scale from 1 (not severe) to 5 (very severe).

Preventive behavior was assessed by eight items questioning about basic protective measures recommended at that time by health authorities: “During the last 7 days, which of the following measures have you taken to prevent infection from COVID-19?” Participants were asked to answer (yes/no) to the following measures: wearing facemasks according to norms and recommendations, ventilating closed spaces, using hydro alcoholic gel or disinfectants for cleaning the hands, washing hands often with soap and water, avoiding busy places, ensuring physical distancing (at least 2 m), avoiding social/family events and wearing a facemask outdoors.

Trust in different sources of information was assessed asking: “How much do you trust information about COVID-19 from the following sources?” (Scientists, health professionals, friends, mass media, internet, social networks, government website and the WHO), answered in a scale from 1 (very little trust) to 5 (a lot of trust).

COVID-19 related health literacy (CHL) was measured following the HLS-EU-Q model (19). It includes a general question: “How easy or difficult is it for you to…?” followed by nine specific tasks related to COVID-19 information access, comprehension, appraisal/evaluation, and application/use. Participants rated their perceived difficulty using a four-category Likert-type scale: very difficult (1), difficult (2), easy (3) and very easy. The CHL questionnaire was recently validated in Spain (20).

### 2.4. Data analysis

All data analyses were carried out separately for each sample. Socio-demographic data and COVID-19 related variables were analyzed using descriptive statistics (frequency, percentages, mean and standard deviation). Two-steps clusters analyses grouped participants according to their reasons for not being vaccinated, using log-likelihood distance between clusters and Schwarz's Bayesian Criterion to determine the optimal number of clusters. To validate the clusters two forward stepwise logistic regression models were performed using clusters as a dependent variable, including socio-demographic and COVID-19 variables. Also, the area under a receiver operating characteristic curve (AUC) was calculated to evaluate the logistic regression predictions. Statistical analysis was executed using SPSS Statistics 27.0.

## 3. Results

A total of 910 non-vaccinated people were included in the social networks sample (SNS) of which 561 (61.6%) were women. The respondents had a mean age of 32 years (standard deviation, SD: 6.1). Most of them (80.1%) had completed university education and were working (77.5%) at the time of the study. The panel sample (PS) was composed by 963 participants, with a mean age of 29.6 years (SD: 6.3) and women represented half of the sample (490, 50.9%). Most of the participants had a university degree (44.9%) and were employed (55.7%). The characteristics of participants are displayed in Table 1.

**Table 1:** Socio-demographic characteristics of the samples.

	**Social networks sample (*****n*** = **910)**		**Panel sample (*****n*** = **963)**	
	***n***	**%**	***n***	**%**
**Age**				
18–29 years	281	30.9	472	49.0
30–40 years	629	69.1	491	51.0
**Sex**				
Man	349	38.4	473	49.1
Woman	561	61.6	490	50.9
**Education level**				
Incomplete primary or less	13	1.4	7	0.7
Primary	6	0.7	186	19.3
Secondary	162	17.8	302	31.4
University	729	80.1	433	45.0
Other/Do not answer	0	0.0	35	3.6
**Employment status**				
Working	705	77.5	537	55.8
Not working^*^	204	22.4	426	44.2
Do not answer	1	0.1	0	0.0
**Type of work**				
With high risk of contagion	137	19.4	90	16.8
With moderate risk of contagion	232	32.9	206	38.4
No risk	335	47.5	240	44.7
Do not answer	1	0.1	1	0.2

^*^Unemployed, student, homemaker.

The main reasons mentioned for not being vaccinated in both samples (Table 2) were believe that the COVID-19 vaccines have been developed too quickly, they are experimental, and they are not safe, answered by 68.7% participants in the SNS and 55.4% in the PS. In addition, 46.3% of respondents in the NHS and 28.5% in the SP consider vaccines to be a business. “I am healthy and do not need to be vaccinated” was answered by 44.3% subjects in the SNS and 22.3% participants in the PS. Reasons related to practical barriers such as the vaccination point is too far away and not knowing what to do to get the vaccine, were reported by <5% of the participants in both samples.

**Table 2:** Reasons for not being vaccinated by sample.

	**Social networks sample (*****n*** = **910)**		**Panel sample (*****n*** = **963)**	
	***n***	**%**	***n***	**%**
Vaccines for COVID-19 have been developed very quickly, they are not safe, they are in the experimental phase	625	68.7	528	54.8
I think vaccines are a business	421	46.3	274	28.5
I am healthy and do not need to be vaccinated	403	44.3	215	22.3
Vaccines are bad for my health	351	38.6	213	22.2
I think the vaccines against COVID-19 do not Work	347	38.1	204	21.2
The coronavirus does not exist, it is a hoax, there is a conspiracy behind it	110	12.1	37	3.8
I don't think I will get infected	105	11.5	50	5.2
I have had COVID-19, I am immune	83	9.1	173	17.9
Medical recommendation of not being vaccinated or health problems	64	7.0	59	6.1
I have a phobia of needles	62	6.8	81	8.4
I don't believe in vaccines in general	55	6.0	50	5.2
I only believe in natural medicine	48	5.3	31	3.2
Religious or ethical reasons	47	5.2	13	1.3
I am pregnant	46	5.1	31	3.2
Distrust in information (it is not clear, it is a lie), in pharmaceutical companies, in the media, in the system, in the government, in the WHO	46	5.1	4	0.4
Concerns about side effects	42	4.6	18	1.8
I am scared because of my legal situation	16	1.8	33	3.4
The vaccination point is too far away	14	1.5	33	3.4
I don't know what I have to do to get the vaccine	6	0.6	29	3.0

Table 3 shows the variables related to preventive behavior, health literacy, trust in information sources, perceived disease severity and vaccination intention in the future. The most frequent preventive behavior in both samples was wearing face masks according to norms (64.1% in SNS and 78.7% in PS); while the least frequent preventive behavior was avoiding social/family events. Concerning health literacy, respondents from both samples found easiest to understand what to do when they are a close contact of a case of COVID-19. Scientists and health workers, followed by internet and friends were the sources of information considered most trustworthy in both samples. The percentage of participants who thought that they would be vaccinated in the future was 11.2% in the SNS and 30.6% in the PS.

**Table 3:** Preventive behavior, health literacy, trust in information sources, perceived disease severity and vaccination intention.

	**Social networks sample**		**Panel sample**	
	***n***	**%**	***n***	**%**
**Preventive behavior (yes)**				
Wearing face masks according to norms	583	64.1%	757	78.7%
Ventilating closed spaces	536	58.9%	516	53.6%
Washing hands often with soap and water	428	47.0%	494	51.3%
Using hydroalcoholic gel or disinfectants	402	44.2%	578	60.0%
Ensuring physical distance	343	37.7%	436	45.3%
Avoiding crowded places	361	39.7%	367	38.1%
Wearing the facemask outside	220	24.2%	366	38.1%
Avoiding social/family events	142	15.6%	194	20.2%
**Do you think you will be vaccinated in the future?**				
No	481	52.9%	250	26.0%
I am not sure	327	35.9%	419	43.5%
Yes	102	11.2%	294	30.6%
	**Mean**	**SD**	**Mean**	**SD**
**Health literacy (1-4)**				
Understanding what to do when you are a close contact of a case of COVID-19	3.2	0.9	3.1	0.8
Follow recommendations on how to protect yourself against coronavirus/COVID-19	2.9	1.0	3.0	0.8
Decide if I should get the coronavirus/COVID-19 vaccine	2.9	1.2	2.7	1.0
Understanding the benefits and risks of being vaccinated against coronavirus	2.6	1.2	2.6	1.0
Finding the information you need about coronavirus/COVID-19	2.6	1.1	2.8	0.9
Understand coronavirus/COVID-19 recommendations and regulations	2.5	1.1	2.6	0.9
Find the information you need about coronavirus/COVID-19 vaccines	2.3	1.1	2.5	0.9
Assess whether the information provided by mass media about COVID-19 is reliable.	2.1	1.2	2.2	1.0
Assess the reliability of media reports about coronavirus/COVID-19 vaccines	2.1	1.2	2.2	1.0
**Trust in information from (1-5)**				
Scientists	2.9	1.4	3.1	1.2
Health professionals	2.4	1.3	3.0	1.2
Internet	2.4	1.3	2.3	1.1
My friends	2.0	1.1	2.4	1.1
The website of the Ministry of Health	1.8	1.2	2.4	1.2
The World Health Organization	1.8	1.1	2.3	1.2
My association	1.8	1.2	2.1	1.1
Social networks (e.g., Facebook, Twitter, YouTube, WhatsApp)	1.7	1.1	1.9	1.0
Television, radio or national press	1.4	0.8	1.8	1.0
My church	1.3	0.8	1.6	1.1
How severe do you think the disease would be if you get infected? (1-5)	3.7	1.3	2.7	1.0

**Figure 1:**
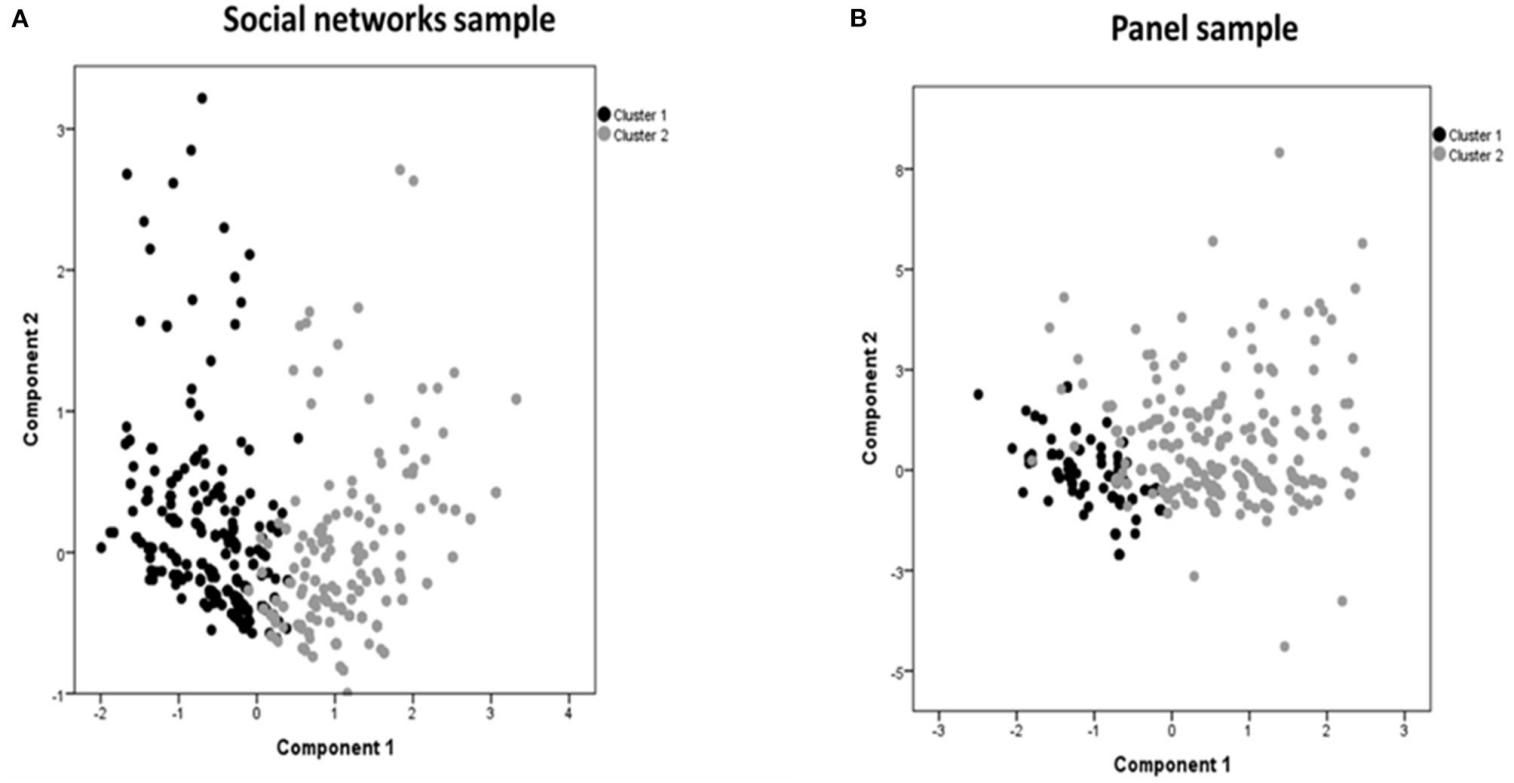
Cluster analysis grouping participants from the social networks sample **(A)** and the panel sample **(B)** according to reasons for not being vaccinated.

Clusters analysis classified participants of each sample into two clusters according to their reasons for not being vaccinated (Figure 1). Cluster 1 gathered participants who answered in a higher proportion than Cluster 2 structural barriers and health-related reasons such as pregnancy, having been previously infected, medical recommendation or other health problems. Cluster 2 grouped participants who mentioned in higher proportion reasons related to distrust on COVID-19 vaccines (safety, efficacy, development, and approval process), conspiracy theories and low risk perception. In the SNS (Figure 1A), Cluster 1 included 562 (61.8%) participants and Cluster 2, 348 (38.2%). In the PS (Figure 1B), Cluster 1 included 400 (41.5%) participants and Cluster 2, 563 (58.5%).

The logistic regression models in both samples (Table 4) show that pertaining to Cluster 2 (vs. Cluster 1) is associated with lower trust in information coming from health professionals (OR: 0.73, 95%CI: 0.61–0.87 for the SNS; OR: 0.72, 95%CI: 0.61–0.85 for the PS); not avoiding social or family events (OR: 0.42, 95%CI: 0.19–0.89 for the SNS; OR: 0.53, 95%CI: 0.33–0.85 for the PS); and unwillingness to be vaccinated in the future (for “yes,” OR: 0.16, 95%CI: 0.06–0.44 for the SNS; OR: 0.11, 95%CI: 0.06–0.2 for the PS).

**Table 4:** Logistic regression models for factors associated to belonging to Cluster 2, in each sample.

	**Social networks sample**			**Panel sample**		
	**OR**	**95% CI**	**P-value**	**OR**	**95% CI**	**P-value**
Living with older people (Ref: yes)	0.445	0.277–0.713	0.001			
**Preventive behavior:**						
Avoiding social/family events (Ref: yes)	0.420	0.199–0.887	0.023	0.533	0.335–0.847	0.008
Physical distancing (Ref: yes)				1.579	1.084–2.301	0.017
Avoiding crowded places (Ref: yes)	0.450	0.290–0.699	<0.001			
**COVID-19 health literacy:**						
Assess the reliability of information coming from the media about coronavirus vaccines				0.687	0.569–0.829	<0.001
How severe do you think the disease would be if you get infected? (1–5)				0.730	0.604–0.881	0.001
**Trust in information from:**						
Health professionals	0.733	0.615–0.874	0.001	0.723	0.614–0.852	<0.001
Social networks				1.271	1.058–1.526	0.010
Internet	1.352	1.163–1.572	<0.001			
The World Health Organization	0.559	0.430–0.726	<0.001			
**Do you think you will get vaccinated in the future? (Ref: No)**						
Not sure	0.300	0.195–0.462	<0.001	0.296	0.177–0.494	<0.001
Yes	0.165	0.062–0.442	<0.001	0.115	0.066–0.201	<0.001

Dependent variable: Cluster 1= 0; Cluster 2= 1. Ref, reference; CI, Confidence interval.

In the social networks sample, other significant variables associated with the probability of being in Cluster 2 were lack of trust in information from the World Health Organization, higher trust in information from internet, not avoiding crowded places and not living with older people, with ORs between 1.35 and 0.44. In the panel sample, Cluster 2 was significantly associated to maintaining physical distancing, higher trust in information from online social networks, lower ability to assess the reliability of media COVID-19 vaccine information and lower perceived severity of the disease, with ORs between 1.58 and 0.69.

The AUC was 0.84 (95% CI: 0.82–0.87) for the social networks sample and 0.77 (95% CI: 0.74–0.81) for the panel sample.

## 4. Discussion

Despite the success of the COVID-19 vaccination campaign in Spain, at the time of this study, almost 10% of the target population was unvaccinated and this percentage increased to around 20% in people aged 18–40 years (6). This is the first nation-wide study in Spain addressing the main reasons for being unvaccinated and its associated factors, using a combination of sampling techniques to ensure access to the intended population (4).

The most frequently argued reasons in both samples were that COVID-19 vaccines have been developed very quickly, they are not safe, or are in an experimental phase. Moreover, thinking that vaccines are a business and that the COVID-19 vaccines do not work were also frequently reported motives for not being vaccinated. These results are in line with previous studies showing that lack of confidence is an important driver of COVID-19 vaccine hesitancy (9, 13–15) including distrust in safety, efficacy and actors involved in vaccine development and administration.

Reasons related to low risk perception (i.e., “I am healthy and do not need to be vaccinated,” “I don't think I will be infected”), pointed also by a large proportion of the participants in both samples, were already found to be associated with low vaccination intention (9, 14). Conspiracy beliefs have also been reported by several authors as drivers of vaccine hesitancy (9, 21–23), but were less frequently mentioned in this study, as well as reasons related to antivaccine arguments such as “I don't believe in vaccines in general” or “I only believe in natural medicine.” Also, motives related with structural barriers (13, 24) were reported by less proportion of participants in this study, maybe due to the structure of the Spanish health system (universal and free) and the efforts implemented to make the vaccine accessible by the Spanish vaccine strategy (3).

The cluster analysis revealed that participants can accurately be classified into two groups according to their reasons for not getting vaccinated. Clusters were similar for the two samples. Both groups referred vaccines safety concerns as the main reason for being unvaccinated. However, Cluster 1 comprised individuals who reported in higher proportion than those in Cluster 2 constraints mainly related to health-related reasons, such as pregnancy, medical recommendation, having been infected and fear of vaccine side effects. The fear of side effects has been found to be one of the most important determinants of reluctance to COVID-19 vaccination (9) and, according to Eberhardt and Ling (13), may be related to concerns that side effects would interfere with work or childcare. COVID-19 vaccination hesitancy in pregnant women was probably due to worries about possible adverse reactions and negative effects on the fetus and breastfeeding that faced many physicians at the beginning of the COVID-19 vaccination (13, 25, 26).

Cluster 2 gathered participants whose reasons centered in distrust on COVID-19 vaccines (information, development, safety and efficacy), conspiracy thoughts (the coronavirus does not exist, the vaccines are a business) and complacency (I am healthy and do not need to be vaccinated). Herrera-Peco et al. (27) analyzed the COVID-19 antivaccination messages on Twitter in Spain and found a mix of conspiracy theory arguments with vaccine manufacturing misinformation. The perception of COVID-19 vaccines as unsafe or experimental has been reported in previous studies, being “concerns about safety/thinking that a vaccine produced in a rush is too dangerous” one of the main reported reasons in other countries (14).

The logistic regression models supported these findings and showed that three factors are consistently associated to Cluster 2 participants, who report distrust, conspiracy, and complacency reasons for vaccination hesitancy (instead of convenience reasons). This group presented a lower trust in information from health professionals, had a lower willingness to get vaccinated in the future, and avoided less social/family events than those in Cluster 1. These results were common to both the social networks and panel samples.

Distrust in healthcare providers has been found to be an important variable that impacts on vaccine hesitancy (7, 10). While healthcare workers are trusted advisors and influencers of vaccination decisions (28), skeptics might perceive them as part of the same system that tries to impose the vaccine. Participants from Cluster 2 had a higher level of trust on information coming from internet or social networks. Studies show the important role that social media have had in spreading conspiracy theories and anti-vaccine messages (27, 29). Moreover, in the panel sample, difficulties in assessing the reliability of the information on vaccines gathered from the media was more present in Cluster 2. Low health literacy has been linked to unwillingness to be vaccinated in USA, together with conspiracy thoughts and misinformation (30). Accessing accurate information and understanding the quality of information about health issues require critical evaluation skills. As misinformation can alter people's decision-making, leading to a self-perpetuating cycle of bad news, efforts must be made to fight fake news about COVID-19 vaccines (31).

Pertaining to Cluster 2 was associated to lower risk perception (severity) and less adherence to some preventive measures in the regression models, such as avoiding social gatherings (in both samples) and avoiding crowded spaces (in the SNS). In the US, conspiracy theories were also associated to lower preventive measures and lower vaccination uptake (17). Health care professionals may also request support and training to fight misinformation and better communicate vaccine characteristics (technologies, approval mechanism, safety and effectiveness) (32, 33), Previous research has indicated that low risk perception is associated not only to low vaccine uptake, but also to less adherence to preventive behaviors, hindering the pandemic control (34, 35).

Individuals in Cluster 1, who claimed in higher proportion reasons related to constraints, were more prone to be vaccinated in the future than those in Cluster 2. Structural barriers (i.e., difficulties to go to the vaccine location) and reasons related to health status who were more frequently mentioned in this group are contextual and may change in the future. A qualitative study described how pregnant, breastfeeding, or receiving fertility treatment woman rather than refusing vaccination for COVID-19 outright, were just delaying it (13).

Organizational aspects of vaccination campaigns have been found to be crucial for vaccination success, including aspects such as the characteristics of the appointment scheduling system, consultation timetables, vaccination waiting times, online booking and recall systems (33). Recently, Tentori et al. (36) showed an increase of COVID-19 vaccine uptake when an individual appointment was assigned with date, time, and location information, along with instructions on how to change the appointment if necessary.

Limitations of this study are related to the sampling procedure of the social networks sample, that was mainly completed by women and highly educated people and therefore findings might not be generalizable. However, it is an adequate way of accessing to groups of population that may be underrepresented in panel studies. In addition, we did not inquire about political factors in this study, which would call for further research.

## 5. Conclusions

Our results suggest that, in Spain, the main reasons for not being vaccinated are related to safety concerns. Communication strategies focused in providing scientifically sound updated messages and addressing misinformation can help to overcome confidence in vaccine safety.

However, people who refused to be vaccinated are a heterogeneous group, with two main sets of reasons: health-related constrains/convenience, and distrust, conspiracy thinking and low risk perception. Low preventive behavior, low health literacy and low risk perception are factors associated with not being vaccinated. It is important to tailor information strategies addressing these associated factors, and to promote information campaigns that provide reliable information and fight fake news and myths. Future vaccination intention differs in both clusters, so these results are important for developing strategies target to increase vaccination uptake for those who do not reject the COVID-19 vaccine completely.

These results may help guiding public health communication in a way that increases vaccine acceptance in the current booster vaccination campaigns and for future health emergencies.

## Data availability statement

The raw data supporting the conclusions of this article will be made available by the authors, without undue reservation.

## Ethics statement

The studies involving human participants were reviewed and approved by the Ethics Committee of the Institute of Health Carlos III (CEI PI 59_2020-v2). The patients/participants provided their written informed consent to participate in this study.

## Author contributions

MF, MR-B, CR-B, and MJF conceived the study. AB and MD designed and implemented the telematic resources. AA analyzed the data. MF, MR-B, and CR-B wrote the initial draft of the manuscript. MJF was involved in funding acquisition and project administration. All authors collaborated in writing, reviewing, and editing the manuscript.

## References

[1] European Centre for Disease Prevention Control (ECDC). Communicable Disease Threats Report. (2021). Available online at: https://www.ecdc.europa.eu/sites/default/files/documents/Communicable-disease-threats-report-04-june-2021-public.pdf (accessed March 4, 2022).

[2] European Commission,. EU Vaccines Strategy. (2021). Available online at: https://ec.europa.eu/info/live-work-travel-eu/coronavirus-response/public-health/eu-vaccines-strategy_en (accessed March 4, 2022).

[3] Amiel S,. How struggling Spain Became One of Europe's Vaccination Champions. EuroNews (2021). Available online at: https://www.euronews.com/my-europe/2021/09/03/how-struggling-spain-became-one-of-europe-s-vaccination-champions (accessed March 4, 2022).

[4] Forjaz MJ Romay- Barja M Falcon- Romero M Rodriguez-Blazquez C. Spain COVID-19 Snapshot MOnitoring (COSMO Spain): Monitoring knowledge, risk perceptions, preventive behaviours, and public trust in the current coronavirus outbreak in Spain. PsychArchives. (2021) 10.23668/PSYCHARCHIVES.4877

[5] Bernaola Iturbe E Giménez Sánchez F Baca Cots M de Juan Martín F Domingo JD Sánchez MG. Criteria for including vaccines in the immunization schedule of the Spanish association of pediatrics. An Pediatr. (2008) 68:58–62. 10.1157/13114473 18194630

[6] Ministerio de Sanidad. Gestión Integral de la Vacunación COVID-19. Informe de actividad diario 24/11/2021. Madrid: Ministerio de Sanidad (2021). Available online at: https://www.sanidad.gob.es/profesionales/saludPublica/ccayes/alertasActual/nCov/documentos/Informe_GIV_comunicacion_20211124.pdf

[7] MacDonald NE. Vaccine hesitancy: definition, scope and determinants. Vaccine. (2015) 33:4161–4. 10.1016/j.vaccine.2015.04.036 25896383

[8] Betsch C Schmid P Heinemeier D Korn L Holtmann C Böhm R. Beyond confidence: development of a measure assessing the 5C psychological antecedents of vaccination. PLoS ONE. (2018) 13:e0208601. 10.1371/journal.pone.0208601 30532274 PMC6285469

[9] Razai MS Chaudhry UAR Doerholt K Bauld L Majeed A. Covid-19 vaccination hesitancy. BMJ. (2021) 373:n1138. 10.1136/bmj.n1138 34016653

[10] Turhan Z Dilcen HY Dolu I. The mediating role of health literacy on the relationship between health care system distrust and vaccine hesitancy during COVID-19 pandemic. Curr Psychol. (2021) 41:8147–56. 10.1007/s12144-021-02105-8 34312580 PMC8295547

[11] Marzo RR Sami W Alam MZ Acharya S Jermsittiparsert K Songwathana K. Hesitancy in COVID-19 vaccine uptake and its associated factors among the general adult population: a cross-sectional study in six Southeast Asian countries. Trop Med Health. (2022) 50:4. 10.1186/s41182-021-00393-1 34983692 PMC8727234

[12] Age G. Hesitant or not? the association of age, gender, and education with potential acceptance of a COVID-19 vaccine: a country-level analysis. J Health Commun. (2020) 25:799–807. 10.1080/10810730.2020.1868630 33719881

[13] Eberhardt J Ling JA. Qualitative exploration of perceptions of the COVID-19 vaccine in the United Kingdom during the later stages of the vaccine rollout. Int J Transl Med Res Public Health. (2022) 6:1–10. 10.21106/ijtmrph.407

[14] Troiano G Nardi A. Vaccine hesitancy in the era of COVID-19. Public Health. (2021) 194:245–51. 10.1016/j.puhe.2021.02.025 33965796 PMC7931735

[15] Centro de Coordinación de Alertas y Emergencias Sanitarias. Actualización no 486. Enfermedad por el coronavirus (COVID-19) Madrid: Ministerio de Sanidad (2021). Available online at: https://www.sanidad.gob.es/profesionales/saludPublica/ccayes/alertasActual/nCov/documentos/Actualizacion_486_COVID-19.pdf

[16] Ministerio de Sanidad,. Gestión Integral de la Vacunación COVID-19. Informe de actividad diario 19/10/2021. Madrid: Ministerio de Sanidad (2021). Available online at: https://www.sanidad.gob.es/profesionales/saludPublica/ccayes/alertasActual/nCov/documentos/Informe_GIV_comunicacion_20211019.pdf

[17] Centro de Coordinación de Alertas y Emergencias Sanitarias,. Actualización no 509. Enfermedad por el coronavirus (COVID-19). Madrid: Ministerio de Sanidad (2021). Available online at: https://www.sanidad.gob.es/profesionales/saludPublica/ccayes/alertasActual/nCov/documentos/Actualizacion_509_COVID-19.pdf

[18] Survey Tool and Guidance: Behavioural Insights on COVID-19 (produced by the WHO European Region). Available online at: https://www.who.int/europe/tools-and-toolkits/who-tool-for-behavioural-insights-on-covid-19 (accessed July 29, 2020).

[19] Sørensen K Van den Broucke S Pelikan JM Fullam J Doyle G Slonska Z. Measuring health literacy in populations: illuminating the design and development process of the European Health Literacy Survey Questionnaire (HLS-EU-Q). BMC Public Health. (2013) 13:948. 10.1186/1471-2458-13-948 24112855 PMC4016258

[20] Falcón M Rodríguez-Blázquez C Fernández-Gutiérrez M Romay-Barja M Bas-Sarmiento P Forjaz MJ. Measuring COVID-19 health literacy: validation of the COVID-19 HL questionnaire in Spain. Health Qual Life Outcomes. (2022) 20:138. 10.1186/s12955-022-02050-5 36167562 PMC9514704

[21] Xiao J Cheung JK Wu P Ni MY Cowling BJ Liao Q. Temporal changes in factors associated with COVID-19 vaccine hesitancy and uptake among adults in Hong Kong: Serial cross-sectional surveys. Lancet Reg Health West Pac. (2022) 23:100441. 10.1016/j.lanwpc.2022.100441 35359914 PMC8961079

[22] Lindholt MF Jørgensen F Bor A Petersen M. Public acceptance of COVID-19 vaccines: cross-national evidence on levels and individual-level predictors using observational data. BMJ Open. (2021) 11:e048172. 10.1136/bmjopen-2020-048172 34130963 PMC8210695

[23] Romer D Jamieson KH. Conspiracy theories as barriers to controlling the spread of COVID-19 in the U.S. Soc Sci Med. (2020) 263:113356. 10.1016/j.socscimed.2020.113356 32967786 PMC7502362

[24] Bates BR Villegas-Botero A Costales JA Moncayo AL Tami A Carvajal A. COVID-19 vaccine hesitancy in three latin american countries: reasons given for not becoming vaccinated in Colombia, Ecuador, and Venezuela. Health Commun. (2022) 14:1–11. 10.1080/10410236.2022.2035943 35164624

[25] Hosokawa Y Okawa S Hori A Morisaki N Takahashi Y Fujiwara T. The prevalence of COVID-19 vaccination and vaccine hesitancy in pregnant women: an internet-based cross-sectional study in Japan. J Epidemiol. (2022) 32:188–94. 10.2188/jea.JE20210458 35095091 PMC8918615

[26] Battarbee AN Stockwell MS Varner M Newes-Adeyi G Daugherty M Gyamfi-Bannerman C. Attitudes toward COVID-19 illness and COVID-19 vaccination among pregnant women: a cross-sectional multicenter study during August-December 2020. Am J Perinatol. (2022) 39:75–83. 10.1101/2021.03.26.21254402 34598291

[27] Herrera-Peco I Jiménez-Gómez B Romero CS Deudero JJ García-Puente M Benítez De Gracia E. Antivaccine movement and COVID-19 negationism: a content analysis of Spanish-written messages on Twitter. Vaccines. (2021) 9:656. 10.3390/vaccines9060656 34203946 PMC8232574

[28] Paterson P Meurice F Stanberry LR Glismann S Rosenthal SL Larson HJ. Vaccine hesitancy and healthcare providers. Vaccine. (2016) 34:6700–6. 10.1016/j.vaccine.2016.10.042 27810314

[29] Ginossar T Cruickshank IJ Zheleva E Sulskis J Berger-Wolf T. Cross-platform spread: vaccine-related content, sources, and conspiracy theories in YouTube videos shared in early Twitter COVID-19 conversations. Hum Vaccin Immunother. (2022) 18:1–13. 10.1080/21645515.2021.2003647 35061560 PMC8920146

[30] Kricorian K Civen R Equils O. COVID-19 vaccine hesitancy: misinformation and perceptions of vaccine safety. Hum Vaccin Immunother. (2022) 18:1950504. 10.1080/21645515.2021.1950504 34325612 PMC8920251

[31] Rief W. Fear of adverse effects and COVID-19 vaccine hesitancy: recommendations of the treatment expectation expert group. JAMA Health Forum. (2021) 2:e210804. 10.1001/jamahealthforum.2021.0804 36218819

[32] Charrier L Garlasco J Thomas R Gardois P Bo M Zotti CM. An overview of strategies to improve vaccination compliance before and during the COVID-19 pandemic. Int J Environ Res Public Health. (2022) 19:11044. 10.3390/ijerph191711044 36078757 PMC9518554

[33] Trucchi C Costantino C Restivo V Bertoncello C Fortunato F Tafuri S. Immunization campaigns and strategies against human papillomavirus in Italy: the results of a survey to regional and local health units representatives. Biomed Res Int. (2019) 4:6764154. 10.1155/2019/6764154 31355274 PMC6637711

[34] de Vries H Verputten W Preissner C Kok G. COVID-19 vaccine hesitancy: the role of information sources and beliefs in Dutch adults. Int J Environ Res Public Health. (2022) 19:3205. 10.3390/ijerph19063205 35328892 PMC8948729

[35] Dryhurst S Schneider CR Kerr J Freeman AL Recchia G Van Der Bles AM. Risk perceptions of COVID-19 around the world. J Risk Res. (2020) 23:994–1006. 10.1080/13669877.2020.1758193

[36] Tentori K Pighin S Giovanazzi G Grignolio A Timberlake B Ferro A. Nudging COVID-19 vaccine uptake by changing the default: a randomized controlled trial. Med Decis Making. (2022) 42:837–41. 10.1177/0272989X221101536 35658775

